# Pathway analysis of the transcriptome and metabolome of salt sensitive and tolerant poplar species reveals evolutionary adaption of stress tolerance mechanisms

**DOI:** 10.1186/1471-2229-10-150

**Published:** 2010-07-17

**Authors:** Dennis Janz, Katja Behnke, Jörg-Peter Schnitzler, Basem Kanawati, Philippe Schmitt-Kopplin, Andrea Polle

**Affiliations:** 1 Forstbotanik und Baumphysiologie, Büsgen-Institut, Georg-August-Universität Göttingen, Büsgenweg 2, 37077 Göttingen, Germany; 2 Institut für Meteorologie und Klimaforschung, Bereich Atmosphärische Umweltforschung, Karlsruher Institut für Technologie (KIT), Kreuzeckbahnstraße 19 82467 Garmisch-Partenkirchen, Germany; 3 Institut für Ökologische Chemie, Deutsches Forschungszentrum für Gesundheit und Umwelt, Helmholtz Zentrum München, Ingolstädter Landstr. 1, 85764 Neuherberg, Germany

## Abstract

**Background:**

*Populus euphratica *is a salt tolerant and *Populus *× *canescens *a salt sensitive poplar species. Because of low transcriptional responsiveness of *P. euphratica *to salinity we hypothesized that this species exhibits an innate activation of stress protective genes compared with salt sensitive poplars. To test this hypothesis, the transcriptome and metabolome of mature unstressed leaves of *P. euphratica *and *P*. × *canescens *were compared by whole genome microarray analyses and FT-ICR-MS metabolite profiling.

**Results:**

Direct cross-species comparison of the transcriptomes of the two poplar species from phylogenetically different sections required filtering of the data set. Genes assigned to the GO slim categories 'mitochondria', 'cell wall', 'transport', 'energy metabolism' and 'secondary metabolism' were significantly enriched, whereas genes in the categories 'nucleus', 'RNA or DNA binding', 'kinase activity' and 'transcription factor activity' were significantly depleted in *P. euphratica *compared with *P*. × *canescens*. Evidence for a general activation of stress relevant genes in *P. euphratica *was not detected. Pathway analyses of metabolome and transcriptome data indicated stronger accumulation of primary sugars, activation of pathways for sugar alcohol production, and faster consumption of secondary metabolites in *P. euphratica *compared to *P*. × *canescens*. Physiological measurements showing higher respiration, higher tannin and soluble phenolic contents as well as enrichment of glucose and fructose in *P. euphratica *compared to *P*. × *canescens *corroborated the results of pathway analyses.

**Conclusion:**

*P. euphratica *does not rely on general over-expression of stress pathways to tolerate salt stress. Instead, it exhibits permanent activation of control mechanisms for osmotic adjustment (sugar and sugar alcohols), ion compartmentalization (sodium, potassium and other metabolite transporters) and detoxification of reactive oxygen species (phenolic compounds). The evolutionary adaptation of *P. euphratica *to saline environments is apparently linked with higher energy requirement of cellular metabolism and a loss of transcriptional regulation.

## Background

Salinization due to sea-level rise, decreased ground water recharge, inappropriate irrigation regimes, and increases in evapotranspiration is a major threat to sustainable land-use [1]. To prevent soil degradation and to maintain productivity in areas affected by salinity, cultivation of salt tolerant plants is required. This applies not only to agricultural crops, but also to woody plants that are gaining importance as renewable resources.

Over the past years, poplar (*Populus *sp.), a fast growing species with high biomass production, has been established as a model organism for tree research [2-4]. Among the species of this genus *Populus euphratica *Olivier occurs naturally in salt afflicted areas [5] and exhibits high salt tolerance [6]. Under saline conditions, *P. euphratica *is able to maintain higher growth rates and higher photosynthetic rates than other poplar species [7]. To gain insight into the molecular basis of its ability to resist salt stress, transcriptional changes were investigated during short-term salt exposure under controlled conditions as well as in long-term salt acclimated mature trees grown in natural environments [8,9]. Previous microarray analyses based on a stress-responsive EST collection of approximately 6340 unique *P. euphratica *genes revealed surprisingly few, i.e., only 22 genes whose transcript abundances were affected by salinity under field conditions [9]. We, therefore, hypothesize that *P. euphratica *developed innate mechanisms to tolerate salt stress that may require no immediate gene regulation.

Salt cress (*Thellungiella halophila*), a close relative of the herbaceous model plant *Arabidopsis thaliana*, occurs in harsh environments. When salt cress was exposed to excess salinity only relatively few stress-responsive genes were detected compared with *Arabidopsis *that exhibited a global defense strategy [10]. Transcriptome and metabolome analyses suggested stress-anticipatory preparedness in salt cress [10,11]. High levels of stress tolerance are the result of evolutionary adaptation. In addition to other possibilities, plants thriving in extreme environments may achieve protection by constitutive activation of stress-related gene networks. As a consequence these regulons can not be detected by classical microarray approaches analyzing transcriptional changes in response to stress.

To elucidate differences in constitutive gene regulation of salt tolerant and sensitive species, direct cross-species comparisons are required. A difficulty, usually limiting straightforward whole genome comparisons between different species, is that for non-model species microarrays are not available and hybridization efficiencies of different species are affected by sequence dissimilarities of bait set (= probe set of the model species on the microarray) and prey set (cDNAs of transcripts of either model or non-model species). In spite of the above problems, cross-species hybridizations have recently been successfully applied to a number of species when their evolutionary distances were close [12]. The problem has also been partially circumvented by disregarding genes with lower transcript abundances because 'apparent decreases' might have been caused by lower hybridization efficiencies, whereas stronger responses between non-model prey and model bait set are indicative for higher transcript abundance [13].

The major goal of our study was to investigate whether *P. euphratica *possesses a preventive stress defense strategy involving constitutive activation of protective pathways. To this end we compared available sequence data of *P. euphratica *genes with those of *Populus *× *canescens *(syn. *P. tremula *× *P. alba *[Aiton] Sm.), a salt sensitive species [14-16]. Since this analysis revealed high nucleotide identity we used Affymetrix GeneChip Poplar Genome Array to examine transcriptional differences of *P. euphratica *and *P*. × *canescens*. The Poplar Genome Array contains 56,055 probe sets (= baits) that can be interrogated, each composed of 11 individual probes. The array covers the whole genome of *P. trichocarpa *(31,999 probe sets based on gene predictions and EST evidence) and contains additionally 23,657 probes sets of 13 further poplar species (based on mRNA or EST sequence data) including *P. euphratica *as well as *P*. × *canescens *[17]. Here, we provide evidence that evolutionary distance of prey and bait sets affect the results but that a condensed data set can be used after application of appropriate quality filters. Key pathways identified by this approach to differ between the two poplar species were validated by targeted biochemical and physiological analyses as well as by non-targeted metabolite profiling applying Fourier Transform-Ion Cyclotron Mass Spectrometry (FT-ICR MS) [18-20]. Metabolome and transcriptome data were combined by pathway analysis tools [21,22]. We provide evidence that energy metabolism, ion compartmentalization, and phenylpropanoid biosynthetic pathways constitute major differences between *P. euphratica *and *P*. × *canescens*.

## Results

### Sequence identity of structural genes of *P. trichocarpa*, *P. euphratica *and *P*. × *canescens *is high

A prerequisite for a transcriptional comparison of *P. euphratica *and *P*. × *canescens *is a high sequence identity of their mRNAs with the probe set oligonucleotides on the microarray, which are mainly based on *P. trichocarpa *genome data [3]. Therefore, we compiled available sequence information for cDNAs of *P*. × *canescens *(Pc), *P. euphratica *(Pe), and *P. trichocarpa *(Pt). We found full-length and partial sequences of 20 genes for all three poplar species, which were used to assess their relationship by analysis of nucleotide identity (Table 1). Identity was usually high with mean values between 95.6 and 97.2%, and no significant differences between the pair wise comparisons of Pc/Pt and Pe/Pt, between Pe/Pc and Pc/Pt or between Pe/Pc and Pe/Pt were found at P ≤ 0.05 (Bonferoni test). However, there are individual examples for low sequence identity, e.g., NdID (NAD^+ ^dependent isocitrate dehydrogenase in Table 1).

**Table 1:** Comparison of sequence data of 20 genes from *P*. × *canescens *(*Pc*), *P. euphratica *(*Pe*) and *P. trichocarpa *(*Pt*)

	-----*Pe/Pt*-----		-----*Pc/Pt*-----		-----*Pe/Pc*-----		----------Public ID----------		
Gene	Ni	al	ni	al	ni	al	*Pe*	*Pc*	*Pt*
AP	97.50%	648	97.00%	668	97.10%	697	AJ777007	CF231430	589502
ATPase	93.80%	145	98.10%	367	93.80%	144	AJ779572	CX655567	821076
BSP	95.50%	334	96.60%	610	94.60%	334	DQ388455	CU233319	687235
FLA12	96.80%	698	95.30%	742	95.20%	665	AJ777975	CF228244	723575
GAST	95.70%	511	96.80%	539	95.50%	508	FJ238511	CF231013	652064
GD	99.10%	645	99.40%	494	96.00%	603	AJ767665	CU223898	832093
GS	99.20%	663	94.10%	642	94.10%	563	AJ768965	DQ855560	565302
Ill3	97.60%	1462	98.40%	1456	96.80%	1443	AJ744952	AJ744953	729069
IPP	97.70%	639	94.10%	422	94.20%	226	AJ774517	CU225654	578868
MADS	99.00%	205	97.90%	573	99.10%	215	AJ780611	CU306852	575376
MCP	97.40%	466	98.00%	556	97.60%	579	AJ774830	CF231502	640384
NdID	93.10%	405	91.60%	419	92.30%	247	AJ774444	CX656537	558763
NhaD1	98.10%	1667	95.00%	220	95.00%	220	AJ561195	*	54522
NifU	97.00%	492	96.70%	492	96.90%	552	AJ775004	CU224355	834330
RGP3	97.90%	438	98.50%	681	97.50%	434	AJ775165	CX659635	673066
RP	95.10%	548	95.80%	618	97.10%	548	AJ775612	CU233448	733792
RPM	99.00%	297	96.70%	426	94.10%	202	AJ770082	CX656156	713972
SIS	98.60%	589	97.30%	559	96.80%	559	FJ238515	FJ238514	560836
TIL	95.80%	671	94.50%	640	96.40%	640	FJ238513	FJ238512	738040
Ubi2	99.00%	312	97.00%	573	96.90%	557	AJ773956	CU233410	664794

Mean ± SD	97.20 ± 1.8%		96.40 ± 1.9%		95.80 ± 1.7%				

*partial sequence, published in [87].

Public ID: accession number for GenBank (*Pc *and *Pe*), respective JGI *P. trichocarpa *project protein id (*Pt*); ni: nucleotide identity; al: length of aligned sequence fragments; AP: Aquaporin (tonoplast intrinsic protein gamma); ATPase: H^+^-transporting ATPase; BSP: Boiling Stable Protein, Chain A;FLA12: Fasciclin-like arabinogalactan-protein; GAST:GAST-like protein;GD: Glycine dehydrogenase; GS: Plastid glutamine synthetase-like; Ill3: IAA-amino acid hydrolase Ill3; IPP: Inorganic pyrophosphatase; MADS: MADS-Box; MCP: Mitochondrial carrier protein; NdID: NAD^+ ^dependent isocitrate dehydrogenase; NhaD1: Na^+^/H^+ ^antiporter type D; NifU: Nitrogen fixation protein NifU; RGP3: Reversibly glycosylated polypeptide 3; RP: Ribosomal protein L34e; RPM: DNA-directed RNA polymerase; SIS: Salt induced serine-rich; TIL: Temperature induced lipocalin; Ubi2: Ubiquitin.

### Detection and evaluation of transcripts differentially expressed between *P. euphratica *and *P*. × *canescens*

**Figure 1:**
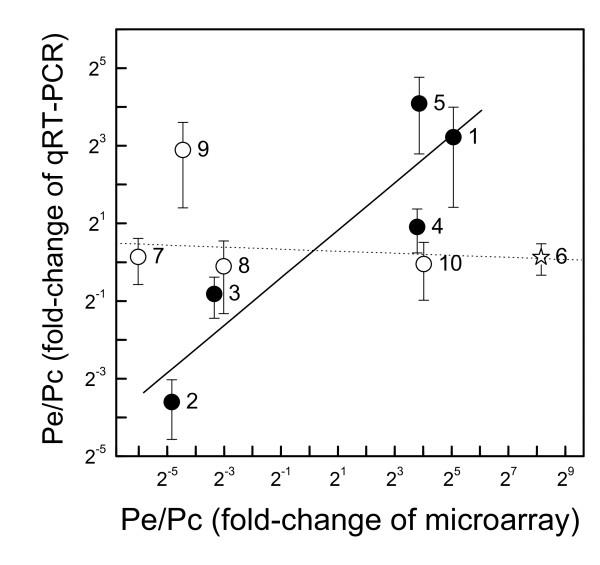
**Correlation of qRT-PCR expression data and microarray signal ratios**. Data show means (SE) for qRT-PCR expression ratios and for signal ratios of microarray data for *P. euphratica/P*. × *canescens*. Transcripts with probe set SDs in the upper 5% quantile are indicated by open symbols, those with lower probe set SDs by black symbols. Star: outlier. The outlier has an SD in the 9% range, which is close to the chosen threshold of 5%. The following genes were included (putative function, Affymetrix probe set ID, JGI gene model for analyzed genes): 1, Gibberellin regulated protein, Ptp.6252.1.S1_a_at, estExt_Genewise1_v1.C_LG_V1745; 2, MADS-Box protein, Ptp.5993.1.S1_a_at, eugene3.00150771; 3, Mitochondrial carrier protein, Ptp.5103.1.S1_at, grail3.0008039502; 4, Lil3 protein, Ptp.4571.1.S1_at, eugene3.01180096; 5, Aquaporin, Ptp.5700.1.S1_s_at, eugene3.00280238; 6, GTP-binding protein, PtpAffx.25286.1.S1_at, estExt_fgenesh4_pg.C_LG_I1368; 7, Nitrogen fixation protein, PtpAffx.1459.1.A1_s_at, estExt_fgenesh4_pm.C_LG_XII0286; 8, Ubiquitin-like protein, PtpAffx.157059.1.S1_s_at, estExt_fgenesh4_pg.C_LG_XIV1291; 9, 1-Aminocyclopropane-1-carboxylate oxidase, Ptp.5158.1.S1_at, estExt_Genewise1_v1.C_1660131; 10, Glycine dehydrogenase, PtpAffx.19705.1.A1_at, estExt_fgenesh4_pm.C_LG_VI0678.

The transcriptomes of mature, fully light-exposed leaves of soil grown *P. euphratica *and *P*. × *canescens *plants were compared by microarray analysis on GeneChip Poplar Genome arrays. In the first step, differentially expressed transcripts were identified by established methods employing significance analysis of microarrays (SAM) and removing transcripts with low signal intensities (Table 2, see Materials and Methods). As cross-species comparisons may be affected by sequence divergence [23,24], we also removed genes whose standard deviation (SD) of mean signal intensities was in the upper 5% quantile. This procedure was based on the following reasoning: since each mean signal is the result of signal intensities of 11 oligonucleotides from different regions of a gene (= 1 probe set), it is likely that sequence divergence will lead to irregular binding of cDNAs within the probe set and thus cause higher SD. High sequence divergence may affect binding efficiencies and thus lead to erroneous results regarding transcript abundance. The validity of this filtering step was corroborated by qRT-PCRs conducted for genes with high and low SDs. Indeed, only those with SDs within the 90 to 95% range showed a significant correlation with the probe sets on the array (R = 0.909, P = 0.032), whereas those within the upper 5% quantile showed no correlation of qRT-PCR and array signal intensities (R = -0.376, P = 0.536, Figure 1).

**Table 2:** Overview of the different filtering steps applied to the list of significant transcripts and the gene universe

Filter number applied	f0	f1	f2	f3	f4	f5
Number of significant transcripts	4557	4305	4080	3568	2379 (2672*)	2246 (2503*)
Size of gene universe	61413	30887	29343	26118	18326	14254

* including transcripts interrogated by probe sets constructed from *P*. × *canescens *sequences that were higher expressed in *P. euphratica*, and transcripts interrogated by probe sets constructed from *P. euphratica *sequences that were higher expressed in *P*. × *canescens*

f0 = raw list: Raw lists are the list of significant genes as calculated by SAM and all probe sets on the microarray; f1 = probe set ID present/absent: filter f1 removed probe sets identified as absent by the MAS 5.0 algorithm; f2 = probe set SD: filter f2 removed probe sets with SD in the upper 5% quantile; f3 = annotation available: filter f3 removed probe sets without annotation; f4: species of origin of probe set: filter f4 removed probes sets biased due to the poplar species used for their construction; f5 = duplicate gene models: filter f5 removed duplicate gene models.

**Figure 2:**
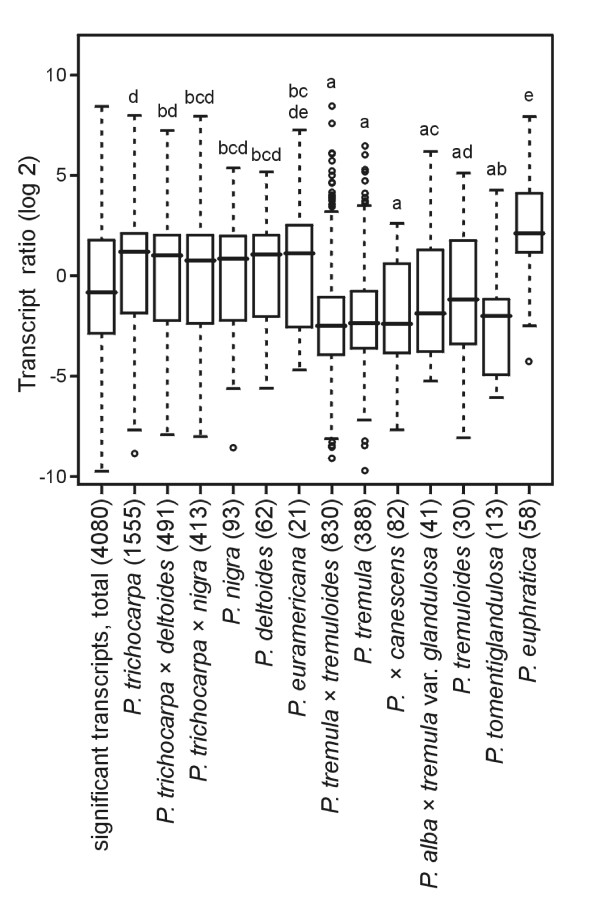
**Transcript ratios of *P. euphratica *and *P*. × *canescens *sorted according to the original poplar species used to construct the probe set on the microarray**. All transcripts with significantly different signal intensities that were detected as present by the MAS 5.0 algorithm and had probe set SDs in the lower 95% quantile were used (f2, Table 2). Signal log ratios were calculated as log-signal_(*P. euphratica*) _- log-signal_(*P*. × *canescens*)_, *i.e*. a positive value denotes a higher apparent expression in *P. euphratica*, a negative value correspondingly a higher apparent expression in *P*. × *canescens*. Sample number of significant transcripts (n) is displayed for each species. Boxes represent the interquartile length (IQL) and median, whiskers extend to the most extreme data point with a maximum length of 1.5 times IQL; outliers are shown as circles;

Despite these efforts, three different groups of transcriptional responses of the Pe/Pc ratio were detected depending on the species used to produce the array probe set (Figure 2). The first and largest group was based on sequences from poplars of the *Tacamahaca *and *Aigeiros *sections (*P. trichocarpa*, *P. trichocarpa *× *deltoides*, *P. trichocarpa *× *nigra*, *P. nigra*, *P. deltoides *and *P. euramericana*). The transcript ratios of Pe/Pc in this group showed a homogenous distribution of increased and decreased transcript ratios (Figure 2). This result is expected if the binding efficiencies of transcripts of the test species *P. euphratica *and *P*. × *canescens *with genes used to construct the probe sets on the array are similar. Only genes of this group were used for GO-term enrichment analyses.

The second group consisted of probe sets based on species of the Leuce section of which *P*. × *canescens *is a member (*P. tremula *× *tremuloides*, *P. tremula*, *P*. × *canescens *[= *P. tremula *× *P. alba*], *P. alba *× *tremula *var. glandulosa, *P. tremuloides *and *P. tomentiglandulosa*). In this group, the distribution of Pe/Pc transcript ratios showed a clear shift towards lower values. Since it is unlikely that the Leuce section contains an overabundance of down-regulated genes this result is probably influenced by sequence divergence resulting in relatively stronger binding of *P*. × *canescens*-derived cDNAs to the probe sets than those of *P. euphratica*, consequently leading to an apparent decrease of the Pe/Pc ratio. We have therefore excluded the genes with decreased Pe/Pc ratio in the Leuce group from further analyses. Vice versa, in the last group, the Turanga section which is formed by *P. euphratica*, the Pe/Pc ratio was increased. Along the same line of argument, the genes with increased Pe/Pc ratios were excluded (Table 2, filter f4*).

Since the goals of this study were functional categorization and pathway analyses, all transcripts without annotation were removed from the list of significant genes as proposed by Falcon and Gentleman [25]. The final list of differentially expressed transcripts contained 2503 genes, of which 1645 had higher transcript abundances in *P. euphratica *and 858 in *P*. × *canescens *than the respective other species (Table 2, Additional data Table S1).

### The transcriptomes of *P. euphratica *and *P*. × *canescens *exhibit differences in transport, energy metabolism and biosynthesis of secondary metabolites

**Figure 3:**
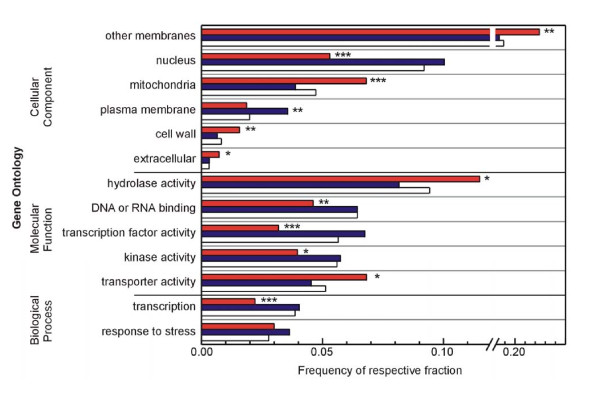
**Over- or underrepresented GO slim categories in *P. euphratica *(red) and *P*. × *canescens *(blue)**. Bars show frequencies of GO slim annotations of genes with significantly higher relative transcript abundance in *P euphratica *(red) or *P*. × *canescens *(blue) compared with the 'gene universe' as defined in table 2 (white). All GO slim categories significantly over- or underrepresented as calculated by a hypergeometric test after Benjamini-Hochberg correction are shown except 'unknown biological processes' (underrepresented in *P*. × *canescens*), 'other enzyme activity' (overrepresented in *P. euphratica*) and 'unknown cellular components' (underrepresented in *P. euphratica*). Data set after filter 5 (table 2) was used. Significant over- or underrepresentation of categories are indicated by * for P ≤ 0.05, ** for P ≤ 0.01 and for P ≤ 0.001.

To characterize differences between the transcriptomes of the two poplar species, we used the GO slim terms of the complex Gene Ontology annotations [26]. A 'gene universe' of 14,254 eligible genes was compiled after appropriate filtering from all 61,413 probe sets on the microarray (Table 2). Significant over- and underrepresentation of GO slim terms for *P. euphratica *and *P*. × *canescens *were determined by a hypergeometric test. In *P. euphratica *GO slim terms associated with transcription and regulation like 'nucleus', 'DNA or RNA binding', 'transcription factor activity', 'kinase activity' and 'transcription' were significantly underrepresented (Figure 3). This suggests that mechanisms to react to alterations of external conditions and to adapt the transcriptome accordingly were less active in *P. euphratica *than in *P*. × *canescens*. Overrepresented GO slim terms in the group of genes with higher expression in *P. euphratica *were 'other membranes' (*i.e*. other than plasma membrane), 'mitochondria', 'cell wall', 'hydrolase activity' and 'transporter activity' (Figure 3). In *P*. × *canescens*, the only significantly overrepresented GO slim term was 'plasma membrane'. Neither of the species showed an over- or underrepresentation of the GO slim term 'response to stress' contrary to our expectation (Figure 3).

**Figure 4:**
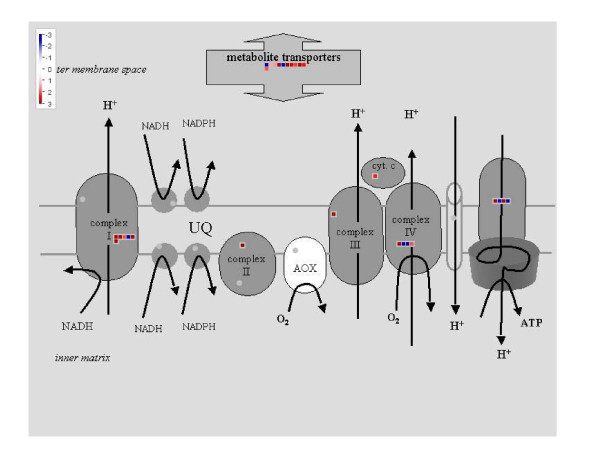
**Mitochondrial electron transport chain and metabolite transporters**. Mapman analysis of the category mitochondria with relative increased transcript abundance in *P. euphratica *(red) and in *P*. × *canescens *(blue).

For more detailed analysis, the best match of the poplar gene models with *Arabidopsis thaliana *was searched, AGI numbers were assigned to the poplar genes, and Mapman was used for a functional classification [27]. In the category 'transport' a number of genes for cation transport with higher transcript abundance in *P. euphratica *than in *P*. × *canescens *were especially conspicuous with respect to the increased salt tolerance of *P. euphratica *(Additional file 1, Table S2). In the category 'cell wall' numerous glycosylases and hydrolase such as putative xyloglucan endotransglycosylases, glycosyl hydrolase family proteins in addition to pectin esterases and fasciclin-like proteins had higher transcript abundance in *P. euphratica *than in *P*. × *canescens *(Additional file 1, Table S2). Analysis of the category 'mitochondria' revealed that most components of the mitochondrial electron transport chain were up-regulated in *P. euphratica *compared with *P*. × *canescens *(Figure 4). Furthermore, out of twelve significantly differentially expressed genes encoding mitochondrial metabolite transporters, ten showed higher transcript abundance in *P. euphratica *than in *P*. × *canescens *(Figure 4).

Among all genes with significant changes that were uploaded into Mapman (n = 2216), the category 'secondary metabolism' was significantly enriched (P = 0.02 after Benjamini Hochberg correction). This result was corroborated using the original GO annotation of the poplar gene models. Both GO terms 'phenylpropanoid' and 'flavonoid biosynthetic process' were significantly enriched in *P. euphratica *compared with *P*. × *canescens *with P = 0.042 and P = 0.046, respectively, after Benjamini Hochberg correction.

### Integration of metabolomic and transcriptomic data on pathway maps and validation by physiological and biochemical analyses

The metabolomes of leaves of the two poplar species were analyzed by Fourier transform-ion cyclotron resonance mass spectrometry (FT-ICR-MS). Identification of ions was performed using 'Mass Translator into Pathways' [MassTrix; 22]. 789 ions were found with had significantly higher peak intensities in *P. euphratica *than in *P*. × *canescens*. To 265 of these ions, a bulk chemical formula could be assigned, of which 75 were annotated in poplar. Where metabolites share an identical bulk formula, multiple alternative annotations may be possible. Therefore, the 75 ions represent 39 different bulk chemical formulas for metabolites with higher abundance in *P. euphratica *than in *P*. × *canescens *(Additional file 1, Table S3). In *P*. × *canescens*, 983 ions with significantly higher peak intensities than in *P. euphratica *were detected. Assignment of a bulk chemical formula was possible for 313 ions, of which 96 with 51 different bulk formulas were annotated in poplar (Additional file 1, Table S3).

Identified and annotated metabolites were automatically mapped to KEGG pathways using MassTrix, simultaneously implementing the transcriptomic data. For this, Enzyme Commission (EC) numbers were obtained for significant genes from the JGI *P. trichocarpa *project [28]. Of the differentially expressed genes, 289 genes with higher expression in *P. euphratica *than in *P*. × *canescens *and 132 genes with higher expression in *P*. × *canescens *than in *P. euphratica *could be annotated by EC numbers and were added to the MassTrix pathway analysis query.

Based on the results of the GO term enrichment analysis that indicated significant effects in secondary metabolism and mitochondrial energy metabolism, five pathways were chosen from the 124 KEGG pathways available for poplar: 'phenylpropanoid biosynthesis', 'flavonoid biosynthesis', 'citrate cycle' with the connected pathways 'glycolysis/gluconeogenesis' and 'starch and sucrose metabolism'.

**Figure 5:**
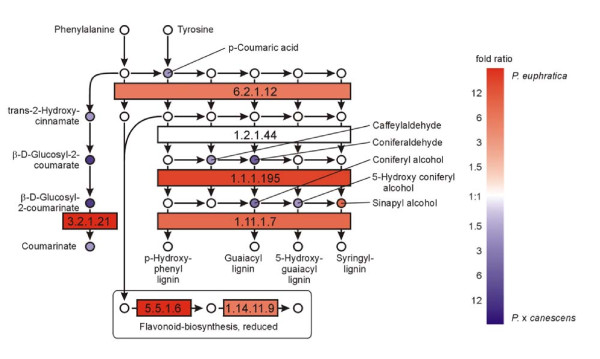
**Analysis of pathways related to phenolics metabolism**. Map displays selected steps from KEGG pathways dpop00940 'Phenylpropanoid biosynthesis' and dpop00941 'Flavonoid biosynthesis'. Colors indicate significant expression, respective metabolite content ratios between *P. euphratica *and *P*. × *canescens*, red indicates higher relative levels in *P. euphratica*, blue in *P*. ×*canescens*. Enzymes are given as EC numbers: 1.1.1.195, cinnamyl-alcohol dehydrogenase; 1.1.1.219, dihydroflavonol 4-reductase; 1.11.1.7, peroxidase; 1.14.11.9, flavanone 3-hydroxylase; 1.2.1.44, cinnamoyl-CoA reductase; 3.2.1.21, beta-glucosidase; 5.5.1.6, chalcone isomerase; 6.2.1.12, 4-coumarate:CoA ligase.

**Figure 6:**
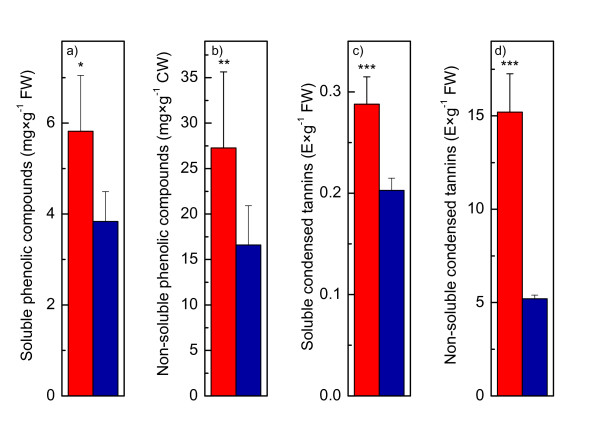
**Phenolic compounds in leaves of P. euphratica (red) and P. canescens (blue)**. Soluble (a) and non-soluble phenolic compounds (b) were determined as catechin equivalents; soluble (c) and non-soluble condensed tannins (d) were determined as extinction units per gram g of fresh weight (n = 5, mean ± SD). Data in (b) are expressed mg per g of cell wall material (CW). Significant differences between the two poplar species are indicated by * for P ≤ 0.05, ** for P ≤ 0.01 and ** for P ≤ 0.001.

The pathways of phenylpropanoid and flavonoid biosynthesis showed higher transcript abundances of enzymes from *P. euphratica *than for those from *P*. × *canescens*, whereas the intermediate metabolites, with the exception of sinapyl alcohol, were usually higher in *P*. × *canescens *than in *P. euphratica *(Figure 5). Since this result may indicate higher flux through the phenolic pathway in *P. euphratica *than in *P*. × *canescens*, which would be expected to result in increased concentrations of end products, we determined soluble and non-soluble phenolics and tannins. In fact, the concentrations of the final products, especially condensed non-soluble tannins, were significantly higher in *P. euphratica *than in *P*. × *canescens *(Figure 6a-d).

**Figure 7:**
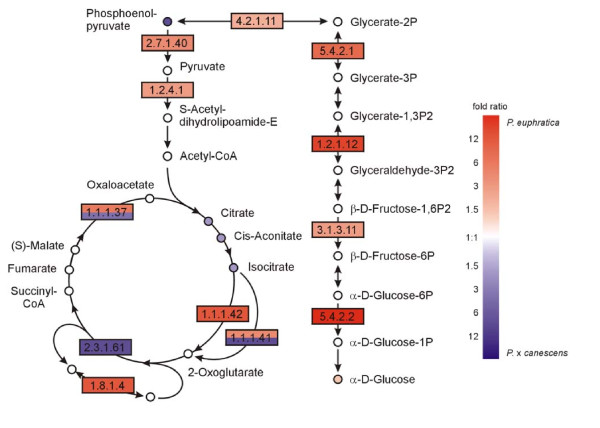
**Analysis of pathways related to energy metabolism**. Map displays selected steps from KEGG pathways dpop00010 'Glycolysis/Gluconeogenesis' and dpop00020 'Citrate cycle (TCA cycle)'. Colors indicate significant expression, respective metabolite content ratios between *P. euphratica *and *P*. × *canescens*, red indicates higher relative levels in *P. euphratica*, blue in *P*. × *canescens*. Enzymes are given as EC numbers: 1.1.1.37, malate dehydrogenase; 1.1.1.42, isocitrate dehydrogenase; 1.2.1.12, glyceraldehyde-3-phosphate dehydrogenase; 1.2.4.1, pyruvate dehydrogenase; 1.3.5.1, succinate dehydrogenase; 2.3.1.12, dihydrolipoamide S-acetyltransferase; 2.7.1.40, pyruvate kinase; 3.1.3.11, fructose-bisphosphatase; 4.1.2.13, fructose bisphosphate aldolase; 4.2.1.11, 2-phospho-D-glycerate hydrolyase; 5.4.2.1, phosphoglycerate mutase; 5.4.2.2, phosphoglucomutase.

Since our transcriptome analysis pointed to higher activity of the mitochondrial electron transport chain in *P. euphratica *than in *P*. × *canescens*, we mapped enzymes and metabolites of the glycolysis and the tricarboxylic acid cycle, necessary to supply the respiratory chain with reductant and localized in the cytosol and the mitochondrial matrix, respectively (Figure 7). While many of the enzymes involved in these pathways were up-regulated at the transcriptional level in *P. euphratica*, only few differences were found regarding the participating metabolites. Among these metabolites most were increased in *P*. × *canescens *compared with *P. euphratica *(Figure 7). Interestingly, the increases of metabolites were observed at the entry point of the TCA cycle (phosphoenol pyruvate, citrate, cis-aconitate and isocitrate), which may indicate lower demand and thus, slower turn over of this pathway in *P*. × *canescens*. This assumption was corroborated by lower dark respiration in *P*. × *canescens *than in *P. euphratica *(Table 3).

**Table 3:** Photosynthesis, respiration and carbohydrates in P. euphratica and *P*. × *canescens*

Parameter	*P. euphratica*	*P*. × *canescens*
Net photosynthesis (μmol CO_2 _m^-2^s^-1^)	2.97 ± 0.58***	4.79 ± 0.30
Transpiration (μmol H_2_O m^-2^s^-1^)	0.79 ± 0.41(*)	1.91 ± 0.09
Dark respiration (μmol CO_2 _m^-2^s^-1^)	1.43 ± 0.05***	0.34 ± 0.39
Glucose (μmol g^-1 ^FW)	9.80 ± 3.15***	4.43 ± 1.52
Fructose (μmol g^-1 ^FW)	0.43 ± 0.06*	0.38 ± 0.02

Photosynthesis and transpiration were determined at 150 μmol m^-2 ^s^-1 ^PAR, a temperature of 26°C and an air humidity of 60%. Dark respiration was measured for 5 min after keeping the plants in darkness for 30 minutes (n = 4, mean ± SD). Glucose and fructose content were determined in mature fully light exposed leaves (n = 5, mean ± SD). Significant differences are indicated by (*) for P ≤ 0.09, * for P ≤ 0.01 and *** for P ≤ 0.001.

**Figure 8:**
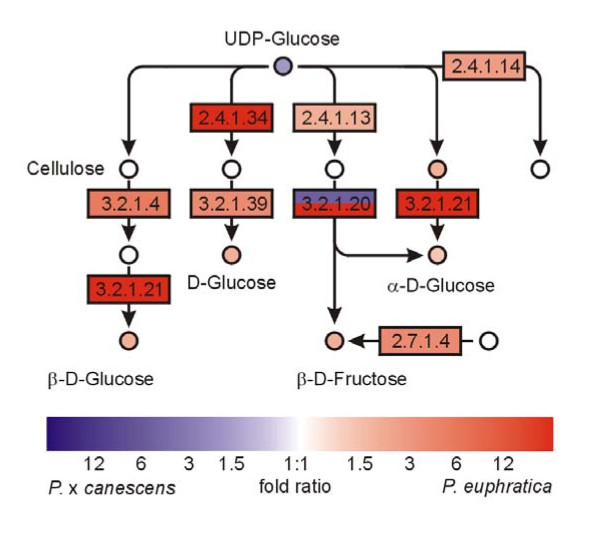
**Pathway analysis of starch and sucrose metabolism**. Map displays selected steps from KEGG pathway dpop00500 'Starch and Sucrose metabolism'. Colors indicate significant expression, respective metabolite content ratios between *P. euphratica *and *P*. × *canescens*, red indicates higher relative levels in *P. euphratica*, blue in *P*. × *canescens*. Two-colored enzymes can occur where different genes models are annotated by the same enzymatic function. Enzymes are given as EC numbers: 2.4.1.13, sucrose synthase; 2.4.1.18, 1,4-α-glucan branching enzyme; 2.4.1.34, 1,3-β-glucan synthase; 3.2.1.2, β-amylase; 3.2.1.3, glucan 1,4-α-glucosidase; 3.2.1.4, cellulase; 3.2.1.20, α-glucosidase; 3.2.1.21, β-glucosidase; 3.2.1.39, glucan endo-1,3-β-D-glucosidase.

Further analyses of carbohydrate metabolism indicated up-regulation of transcripts for genes involved in starch and carbohydrate metabolism in *P. euphratica *compared with *P*. × *canescens *(Figure 8). In addition, pathway analysis of metabolome data pointed to increased concentrations of glucose and fructose in *P. euphratica *relative to *P*. × *canescens *(Figure 8). Independent biochemical carbohydrate analyses revealed that *P. euphratica *contained double the glucose concentrations of *P*. × *canescens *(Table 3). Higher activity of sugar metabolism can also be inferred from six carbohydrate transporters with increased transcript abundance in *P. euphratica *compared with *P*. × *canescens*, whereas no transporters of this class were found to be increased in *P*. × *canescens *(Additional file 1, Table S2). The net CO_2 _assimilation rates were, however, lower in *P. euphratica *than in *P*. × *canescens *(Table 3). It is likely that this difference was mainly caused by Rubisco activase, a chaperone that maintains Rubisco activity [29], which showed significantly lower transcript abundance in *P. euphratica *than in *P*. × *canescens *(Additional file 1, Table S2)

Since carbohydrates also play roles in salt tolerance by counteracting osmotic stress, transcriptional and metabolomic differences for 'minor carbohydrates' according to Mapman categories were also studied. The raffinose and myo-inositol pathways with enzymes leading to formation of galactinol, raffinose and stachyose as well as to myo-inositol were transcriptionally increased in *P. euphratica *compared with *P*. × *canescens *(Additional file 1, Table S2). Increased concentrations of myo-inositol and of raffinose were detected in *P. euphratica *compared with *P*. × *canescens *(Additional file 1, Table S3).

## Discussion

### Direct transcriptome comparisons of different poplar species are affected by their phylogenetic distances

In spite of its regular application, correct use of cross-species hybridization on microarrays is still an intensively discussed topic. Mismatch as a result of single nucleotide polymorphisms can decrease the signals of single probes and influence the mean probe set signal leading to a higher standard deviation as shown here and in cases of strong sequence divergence to systematically lower signal intensities [23,30]. To avoid species-related bias when comparing the transcriptomes of *Arabidopsis thaliana *and its close relative the salt tolerant cress *Thellungiella halophila *on an *Arabidopsis *cDNA microarray, only genes with higher signal ratios of *Thellungiella/Arabidopsis *were considered [13]. However, this may lead to loss of important information with respect to the biological differences.

Recently, Renn *et al. *[31] showed for different cichlid fish species that the consistency of cross-species hybridization microarray results increased with decreasing phylogenetic distance of the analyzed species. For species with a time of genetic divergence of less than 10 million years (Mya) ago, transcription profiling gave most robust results, but divergence times of greater than 65 Mya were still acceptable. The genus of *Populus *diverged from *Salix *around 60 to 65 Mya ago and evolution of *Populus *proceeds relatively slowly, approximately at one-sixth of the rate of *Arabidopsis *[3]. In line with this notion sequence identity of structural genes between *P*. × *canescens*, a species from the oldest section Leuce and *P. trichocarpa*, which is member of the relatively young Tacamahaca section [32], was not significantly different from that between *P. euphratica *in the section Turanga, which assumes a phylogenetically intermediate position, and *P. trichocarpa*. Still, a clear species-related bias for signal ratios was observed that reflecting the genetic phylogeny of different poplar species. We, therefore, conclude that interspecific transcriptome comparisons using Poplar Genome Array between species from different sections require the exclusion of false positives by appropriate filtering procedures, whereas comparisons within a phylogenetic section are unproblematic.

### Pathway mapping reveals differences in energy and secondary metabolism between *P. euphratica *and *P*. × *canescens*

Applying tools of systems biology, we demonstrated that pathways of energy metabolism and for the production of secondary metabolites constituted major differences between *P. euphratica *and *P*. × *canescens*. It has been demonstrated for species in different kingdoms including poplars that transcript-level variation is under genetic control and heritable [33-37]. The identified differences between the transcriptomes of a salt tolerant and a susceptible poplar species reflect their inherited molecular phenotypes since developmental and environmental factors, that are also influencing gene regulation, were excluded. However, in contrast to our expectation no evidence for general increases in typical pathways mediating stress tolerance were found in *P. euphratica *compared with *P*. × *canescens*. Similarly, transcriptional comparisons of the salt tolerant *T. halleri *and the susceptible *A. thaliana *did not show activation of general stress responses but rather pointed to a precise defence strategy [10,38,39].

It is debatable if all observed differences between *P. euphratica *and *P*. × *canescens *were specifically related to their differences in salt susceptibility. For example, increases in phenolics induced by chemical effectors that resulted in ameliorative effects on salt stressed plants also correlated with increases in other antioxidative compounds [40]. Therefore, the contribution of phenolic compounds to improve salt persistence is still elusive and requires further investigation. However, it is well known that phenolic compounds have antioxidant properties [41]. Since excess salinity causes oxidative stress [6], it is likely that phenol-based compounds prevent negative effects of salt-related oxidative stress by scavenging free radicals, thereby, contributing to maintain cell and tissue integrity. Notably, the phenylpropanoid metabolism was also among the significantly increased pathways identified in a comparison of drought susceptible and tolerant poplar species [37]. Since *P. euphratica *is not drought tolerant [42] we suggest that phenolic compounds do not constitute a primary line of defence to prevent osmotic imbalance but that their enrichment may enhance stress tolerance by downstream effects such as affording increased radical scavenging.

Further major transcriptional differences between the two species were related to carbohydrate metabolism and confirmed by analysis of carbohydrate concentrations. Enhanced glucose, fructose, raffinose, and myo-inositol levels in *P. euphratica *leaves suggest that this species is pre-adapted to osmotic stress. An interesting feature is that the elevated carbohydrate concentrations do not necessitate further increases in osmolytes upon exposure to excess salinity. On the contrary, we have previously shown that osmotic adjustment, which is required when sodium accumulates, is achieved by decreases in carbohydrates, especially that of glucose [43]. Furthermore, some classes of osmolytes such as sugar alcohols ameliorate the negative consequences of high ionic strength by preventing unfolding and precipitation of proteins [44-46]. Further support for the importance of chaperones for salt preparedness comes from transcriptome analyses showing that transcript levels of cyclophilins (category 'cell organisation'), heat shock proteins and chaperones (category stress) were increased in comparison with *P*. × *canescens *(Additional file 1, Table S3). It is probably an advantage of *P. euphratica *that protective proteins and metabolites are already in place before the stress starts. Up-regulation of these pathways is found in *P*. × *canescens *only upon salt exposure [47] and, therefore, the protective effect in salt sensitive species may lag too much behind to prevent effectively salt imposed injury.

### Salt preparedness involves constitutively high expression of ion carriers

A striking and important finding is the overrepresentation of transport systems in *P. euphratica *compared to *P*. × *canescens *(Figure 3). A closer inspection of this category shows the presence of ABC transporters, metabolite transporters, sugar transporters and transporters with known functions in salt tolerance such as aquaporins, Ca^2+^, Na^+^, and K^+ ^transporters [48]. For instance, gating of aquaporins is important to maintain the water balance under stress [49]. In several species, including field grown *P. euphratica*, the expression of aquaporins decreased under salt stress [9,50,51], whereas overexpression of aquaporins increased plant vigour only under non-stressed conditions [52]. The activation of water channels in non-stressed *P. euphratica *suggests higher water supply under normal conditions. This conclusion is corroborated by a higher ratio of transpiration to photosynthesis in *P. euphratica *than in *P*. × *canescens *supporting that *P. euphratica *is a phreatophytic species [53].

Maintenance of calcium and potassium supply are crucial features of salt tolerance [48]. In *P. euphratica*, K^+ ^efflux was lower than in a salt sensitive poplar species involving Na^+^-induced down regulation of potassium outward rectifying channels and nonselective cation channels and regulated by Ca^2+ ^[54,55]. In our study, putative Ca^2+^-activated outward rectifying K^+ ^channels showed higher expression in *P*. × *canescens *than in *P. euphratica*, whereas several systems for K^+^-influx were increased in *P. euphratica*. The expression and response to salt stress of a large range of K^+ ^channels has been screened in *P*. × *canescens *[16]. In leaves, none of these transport systems increased in response to salinity and some were not detectable such as the stelar K^+^-outward rectifying channel (SKORC) that mediates the delivery of K^+ ^from stelar cells to the xylem in the roots towards the shoot. SKORC was neither expressed in leaves *Arabidopsis thaliana *[56], whereas considerable activation was found in *P. euphratica *leaves. The most important K^+^-transporter with respect to salt tolerance is probably HKT1, which was among the most highly upregulated genes (92 fold) in *P. euphratica *compared with *P*. × *canescens*. HKT1 is a high affinity K^+ ^uptake transporter that facilitates Na^+ ^homeostasis [57,58]. In *Arabidopsis thaliana *AtHKT1;1 controls the rate of Na^+ ^transport from root to shoot by the retrieval of Na^+ ^from the xylem in the roots [59] and therefore is a key factor in determining salt tolerance in various species [60]. In *P. euphratica *sodium transport from root to shoot is also more strongly restricted than in salt sensitive species but the molecular basis of this effect is not yet known [61]. The strong transcriptional activation of *PeHKT1 *suggests that this transporter is crucial for the control of Na^+ ^in *P. euphratica *leaves.

NHX-type antiporters, which are involved in Na-extrusion from the cytosol back into the apoplast, or into endosomal compartments or the vacuole [62,63], were not identified in our analysis. However, *NaHD1 *was increased in *P. euphratica *compared with *P*. × *canescens*. NaHD1 is a sodium proton antiporter, whose heterolog expression in *E. coli *that was lacking its two sodium/proton antiporters NahA and NahB restored salt tolerance [64]. The activity of PeNaHD1 is pH dependent and functions only at low pH [64]. Maintenance of its activity therefore requires a sufficiently large proton gradient. Our comparative analysis revealed increased transcript levels of H^+^-ATPases suggesting maintenance of a higher proton gradient in *P. euphratica*. Since permanent activation of proton pumps is ATP-consuming, a higher energy demand is expected for *P. euphratica*. It is, therefore, reasonable to assume that the observed increases in mitochondrial respiration in *P. euphratica *compared with *P*. × *canescens *are required as driving force for a tighter control of ion compartmentalization.

## Conclusions

We showed that direct comparisons of the transcriptomes of different poplar species on Poplar Gene Array are influenced by their phylogenetic distance, if the species are from different sections as applies for the salt sensitive *P*. × *canescens *(Leuce) and the salt tolerant *P. euphratica *(Turanga). This influence can be reduced by application of appropriate filters. Using filtered transcriptome data, pathways for the production of secondary metabolites, and carbohydrates as well as a significant enrichment of genes in the categories 'transporters' and 'mitochondria' were identified that were activated in *P. euphratica *compared with *P*. × *canescens*. This interpretation was corroborated by a combination of metabolomic analyses and targeted biochemical and physiological measurements suggesting that higher metabolic flux through the pathways resulted in the observed increases of phenolic end products and carbohydrates in *P. euphratica *compared with *P*. × *canescens*. Tolerance mechanisms for excess salinity in *P. euphratica *are obviously relying on stronger pre-formed osmolyte and chaperone abundance and on tighter control of ion compartmentalization requiring increased energy allocation.

Underrepresentation of GO terms related to signaling and transcription suggest that mechanisms responsible for gene regulation and transcription were less active in *P. euphratica*. Since previous analyses revealed only moderate transcriptomic changes in response to stress [9], we propose that the underside to a high level of stress preparedness as a result of adaptation to a specific ecological niche is a loss of flexibility and adjustability of the transcriptome to environmental cues.

The results of our study may also have implications for wood production. Poplars are fast fast-growing tree species that constitute an important resource for woody biomass. However, expansion of tree cultivation will only be possible on marginal or polluted soils that are not suitable for food production [65]. For this purpose stress tolerant tree species are required. The results of this study laid a foundation for understanding tree salt tolerance at the level of system biology. This knowledge can be used for tree improvement by biotechnological methods or conventional breeding.

## Methods

### Plant culture

Plantlets from *P*. × *canescens *(*P. alba *× *tremula*) clone INRA717 1-B4 and *P. euphratica *clone B2 from the Ein Avdat valley in Israel were multiplied by *in vitro *micropropagation [66], transferred to aerated hydroponics using Long Ashton nutrient solution [67] for acclimation to greenhouse conditions, subsequently potted into soil (Frühstorfer Erde, Lauterbach-Wallenrod, Germany) and grown for 3 months in a greenhouse at 20 to 25°C, with a relative air humidity of 40 to 60% and additional 16 h of light (neon lamps: L58W/25 and 58W/840, Osram, Munich, Germany, and TLD 58W/840 Philips, Amsterdam, Netherlands) between 6:00 a.m. and 10:00 p.m. to reach a continuous photosynthetic active radiation (PAR) of 150 μmol m^-2^s^-1^. Fully expanded leaves were harvested at a stem height of approximately 70 cm (upper third of the stem) after four h in the light period. The leaves were shock-frozen in liquid nitrogen and kept at -80°C until further analysis.

### Gas exchange

Gas exchange (net photosynthesis, respiration, and transpiration) was measured (1 min means) with a portable gas exchange system (HCM-1000 or GSF-3000, Walz, Effeltrich, Germany) under ambient conditions. For analysis of dark respiration plants were connected to the system and kept for 30 min in darkness prior to analysis. For each plant (n = 5) the gas exchange values of 5 min were averaged.

### FT-ICR-MS measurements

Of each species, 5 biological replicates were measured. Frozen leaf tissue was ground in a ball mill (Retsch, Haan, Germany). Fine powder was diluted in methanol to a methanolic concentration of 70% to give highest ion density inside the electrospray, without eliminating those neutrals which are highly water soluble. High-resolution mass spectra were acquired on a Fourier Transform Ion Cyclotron Resonance Mass Spectrometer (APEX Qe, Bruker, Bremen, Germany) equipped with a 12-T superconducting magnet and an Apollo II Electrospray (ESI) source. The ionization source was run in the negative operation mode to generate mono charged negative ions. Thus, all important multi-functional organic compounds which bear at least one acidic site could be deprotonated to give rise to a detectable anion in the mass spectrometer [18]. Each sample was introduced into the ionization source at a flow rate of 2 μL min^-1 ^by a microliter pump with a nebulizer gas pressure of 20 psi and a drying gas pressure of 15 psi (heated to 200°C). Each sample was measured three times and the signal intensities of each detected ion were averaged before running the multi-dimensional statistical data analysis.

Spectra were externally calibrated on clusters of arginine (10 mg l^-1 ^in methanol); calibration errors in the relevant mass range were always below 0.05 ppmv. The spectra were acquired with a time domain of 1 Megaword with a mass-to-charge ratio (m/z) range of 146-2000. The spectra were zero filled to a processing size of 2 Megawords. Before Fourier transformation of the time-domain transient, a sine apodization was performed. No fragmentation experiments were performed in this study. Thus, the whole mass range could be scanned, and 300 scans were summed on in each acquisition. The ion accumulation time in the ion source was set to 0.1 s.

FT-ICR mass spectra were exported to peak lists at a signal to noise ratio (S/N) of two. From those lists, High to Low signal intensity and Low to High signal intensity profiles were obtained by use of the Hierarchical Clustering Explorer HCE Version 3.0 [68]. Ions with sharp differences between the acquisitions of the two species were highlighted as High to Low and Low to High signal intensity profiles. The m/z values of all ions were clustered by the use of the average linkage method with similarity/difference measure type: Euclidean distance. The search method is model-based and the used distance measure was Pearson's correlation coefficient of 0.8. For all obtained hits, t-tests were calculated to validate a statistical significance at α = 0.1.

Metabolite identification and annotation of the m/z values was performed *via *the MassTRIX web site with the following parameter settings: 'Scan mode negative ionisation (correct for H^+ ^loss)', 'Max. error 1.0 ppm', 'Database KEGG with isotopes', 'Organism *Populus trichocarpa*' [22]. Identified compounds and differentially expressed genes added to the query as EC numbers were automatically mapped to KEGG pathways using KEGG/API [69]*via *MassTrix. Metabolites with the same total formula could not be distinguished.

### Analysis of soluble and cell wall bound phenolics

Frozen leaf tissue was ground in a ball mill (Retsch, Haan, Germany). Fine powder (60 mg per sample) was extracted with 2 ml of 50% methanol in an ultrasonic bath (60 min, 40°C; Sonorex Super RK 510 H, Bandelin electronics, Berlin, Germany). The extract was centrifuged, the pellet was extracted once again and the supernatants were combined for analysis of soluble phenolics photometrically with the Folin Ciocalteus method [70]. Catechin (Sigma-Aldrich, Deisenhofen, Germany) was used for calibration and the phenolic concentrations were expressed as catechin equivalents.

To determine cell wall bound phenolics, the pellet was washed with n-hexane, dried, and weighed. This pellet representing cell walls was extracted with 1 M NaOH as described earlier [70]. The extracted phenolic compounds were measured with the Folin Ciocalteous reagent as above.

### Analysis of soluble and non-soluble condensed tannins

Condensed tannins (proanthocyanidin) were determined after [71]. For extraction of soluble tannins, plant materials ground to a fine powder were extracted with 50% methanol, centrifuged, the pellet was re-extracted and the extracted solutions were combined. Aliquots were mixed with a reagent (1.6% methanol, acid butanol [5% concentrated HCl in butanol (v/v)] and 0.2 ml of 2% FeNH_4_(SO_4_)_2_·12H_2_O in 2 M HCl [FeNH_4_(SO_4_)_2_·12H_2_O/HCl = w/v], incubated at 95°C and used to measure the extinction at 550 nm. The amount of tannins was expressed as absorbance per dry mass.

To determine non-soluble tannins, the pellet was lyophilized, weighed and resuspended in 1 ml of 100% methanol. The slurry was combined with acid butanol reagent and processed as above.

### Soluble carbohydrates

Frozen tissues were ground to a fine powder with a ball mill (Retsch, Haan, Germany) pre-cooled in liquid nitrogen and extracted in DMSO/HCl (dimethylsulfoxid:25% HCl = 80:20 (v:v)) at 60°C for 30 min. The mixture was centrifuged and aliquots of the supernatant were mixed with 0.2 M citrate buffer (pH 10.6). This extract was mixed with a reaction mixture (0.75 M triethanolamine-buffer, 4 mM NADP, 10 mM ATP, 9 mM MgSO_4_, pH 7.6) for enzymatic determination of soluble sugars as described previously [72]. Glucose was measured after addition of hexokinase (3 mg ml^-1^, Roche Diognostics GmbH, Mannheim, Germany), fructose after addition of phosphoglucose isomerase (10 mg ml^-1^, Roche Diognostics GmbH, Mannheim, Germany) and sucrose after incubation with β-fructosidase (5 mg ml^-1^, Roche Diognostics GmbH, Mannheim, Germany). Calibration curves were produced with glucose, fructose and sucrose. The sucrose content of the extracts was below the detection limit of the method.

### In silico sequence analysis

DNA sequences of twenty genes were compared between *P*. × *canescens*, *P. euphratica *and *P. trichocarpa*. For five genes, full length sequences were available for *P. euphratica *and *P*. × *canescens *cDNA ('IAA-amino acid hydrolase', Ill3 [[73] GenBank:AJ744952, GenBank:AJ744953]; 'Na/H antiporter type D', NhaD1 [[74] GenBank:AJ561195]; 'salt induced serine-rich' and 'temperature induced lipocalin', SIS and TIL [[74] GenBank:FJ238515, GenBank:FJ238514 and GenBank:FJ238513, GenBank:FJ238512]; 'gibberellin regulated protein', GAST, [GenBank:FJ238511, GenBank:CF231013]). Criterion for the selection of further genes was their representation by probe sets on the microarray and the availability of sequence data for *P. euphratica *and *P*. × *canescens *in public databases. Probe sets both with and without significantly different signal intensities between *P. euphratica *and *P*. × *canescens *were randomly chosen, and sequence data for the corresponding *P. trichocarpa *gene model retrieved from the *Populus *genome project (Table 1) [28]. Because most sequence data is available from EST datasets and thus as cDNA sequences, open reading frame (ORF) sequences of the *P. trichocarpa *gene models were used to screen the GenBank database for homologs in *P. euphratica *and *P*. × *canescens *by using the Megablast algorithm [75]. Pair wise alignments and base identity calculations of the cDNA sequences were generated with the GeneDoc software [76]. An ANOVA was conducted to test for significant differences between the sequence identity values at α = 0.05.

### RNA extraction

Leaf material of 3 plants was pooled. Three biological replicates per poplar species were analyzed (i.e. a total of 9 nine plants per species was included). Frozen leaf tissue was milled in a ball mill (Retsch, Haan, Germany). Total RNA was extracted from 500 mg of frozen plant powder according to Chang *et al. *[77] with minor modifications: No spermidine was applied in the extraction buffer, and 2% β-mercaptoethanol was used. RNA was additionally purified using an RNeasy Mini Kit (Qiagen, Valencia, CA). Total RNA yield and purity were determined spectrophotometrically (BioPhotometer, Eppendorf, Hamburg, Germany) at A_260 _and A_280_. RNA integrity was assessed on an Agilent 2100 Bioanalyzer (Agilent, Santa Clara, CA) at the Microarray Facility (Tübingen, Germany).

### Microarray analysis

Of each species, 3 biological replicates were analyzed on the GeneChip^® ^Poplar Genome Array (Affymetrix, Santa Clara, CA). Synthesis of one-cycle cDNA and biotin-labelled cRNA, fragmenting of cRNA, hybridization to the Poplar Genome Array, washing, staining and scanning was performed as stated by Affymetrix (GeneChip^® ^Expression Analysis Technical Manual) at the Microarray Facility (Tübingen, Germany). Data were deposited at ArrayExpress [EMBL: E-MEXP-1928].

Statistical analysis of the raw signal intensity data was conducted using the following functions from packages released by the bioconductor project, implemented in R [78,79]. Background correction, quantile normalization and summarization of the Affymetrix CEL output files resulting in a raw list of normalized genes was computed by robust multi-array average (RMA) using the 'rma' function from the 'affy' package [23]. Statistical testing for differentially expressed genes was performed on this raw list with the 'sam' function from the 'siggenes' package [80]. In the SAM analysis, Δ was chosen to obtain FDR = 0.05. Annotation of the genes was carried out via the PopArray-Database [81]. Gene Ontology (GO) terms were matched *via *The Arabidopsis Information Resource (TAIR) [82].

Gene lists were subjected to several filtering processes after [25]: genes that were not expressed were removed by calculating Affymetrix' MAS 5.0 change calls using the 'mas5calls' function from the 'affy' package; only genes with two or three 'present' calls in any one of the two species were considered. Probe sets presumably affected by differences in binding efficiencies were removed. For this purpose, SDs of prey signals were calculated for each bait set on a microarray and then averaged over all six microarrays for each gene. The resulting data were compared with the range of the SDs of all prey set signals. A gene was removed from the list when its prey signal SD was in the upper 5% quantile of all 'present' bait set SDs on the microarray. The setting of the threshold for this filter affects the stringency of the results. For the purpose of this study the upper 5%-quantile was used.

Probe sets for which no annotation was available were removed. In cases where multiple probe sets were matched with the same JGI *P. trichocarpa *gene model, duplicate probe sets were removed so that each gene model was represented only once. The probe set with the lower p-value in the SAM analysis was used for further analysis if both genes showed the same direction of change in gene expression. Probe sets annotated by the same gene model with contrasting expression ratios were removed.

### Bioinformatic analyses

For evaluation of over- and underrepresented GO slim terms, the frequencies of annotations within list of significant genes were compared to the frequencies within the defined 'gene universe'. Statistical testing was conducted by calculating the cumulative hypergeometric distribution function, using the 'phyper' function in R. Benjamini-Hochberg correction was applied to the resulting p-values using the 'p.adjust' function.

For statistical analysis of overrepresentation with 'The Ontologizer' [83], a gene ontology file for *Populus *was adapted. In this file, all JGI *Populus trichocarpa *gene models represented on the Affymetrix GeneChip were linked to the GO identifier of their closest *Arabidopsis *matches as listed by TAIR [82]. The 'population' of the Ontologizer conforms with our 'gene universe', whereas the two lists of genes higher expressed in one of the poplar species each correspond to a 'study set'. As parameter settings, term-for-term analysis with Benjamini-Hochberg correction was used.

### qRT-PCR

For quantitative Real-Time PCR (qRT-PCR), primer pairs were designed for actin 9 [GenBank:AJ778775 (*P. euphratica*) and GenBank:CX656348 (*P*. × *canescens*)] as a reference gene and 10 transcripts that were differentially expressed in *P. euphratica *and *P*. × *canescens *(Additional file 1, Table S4). All primers met the following conditions: 1) 100% sequence identity with both *P. euphratica *and *P*. × *canescens *cDNA; 2) fragment lengths between 90 and 180 base pairs (bp); 3) calculated salt-adjusted melting temperatures T_m_(salt) between 58 and 62°C. Primer design was performed with the Oligo Explorer, and suitable primers were tested for similar T_m_(salt), primer dimers and primer loops by Oligo Analyzer (both Gene Link, Hawthorne, NY, USA).

Total RNA was DNAse treated with a Turbo DNA-free kit (Ambion, Austin, TX) and transcribed to cDNA with a RevertAid™ First Strand cDNA Synthesis Kit (MBI Fermentas, St. Leon-Rot, Germany). For each gene, three technical repeats were analyzed for three biological samples. The qRT-PCR was performed on an iCycler (Bio-Rad, Hercules, CA). 250 ng cDNA were used in a 25 μl reaction with 1 × ABsolute qPCR SYBR Green Fluorescein Mix (ABgene, Surrey, UK; including Thermo-Start DNA Polymerase) and 10 μM primer. First denaturation and activation of the Taq-polymerase occurred at 95°C for 15 min, followed by 45 cycles of denaturation at 95°C for 15 s, annealing at 56°C for 30 s and elongation at 72°C for 30 s.

qRT-PCR outputs of three biological replicates per species and gene were analyzed using the MyiQ software (Bio-Rad, Hercules, CA). Primer specificity was assessed by melting curve analysis. Statistical analysis of expression ratios and standard error was conducted using a pair wise fixed reallocation randomization test implemented in Excel with the Relative Expression Software Tool (REST)-384 [84,85]. All primer pairs showed less than 5% differences in PCR efficiency between *P. euphratica *and *P*. × *canescens*.

### Statistical analyses

Data are shown as means (± SE). Five to six plants were analyzed per species. Statistical analysis (ANOVA) was performed using Statgraphics (Centurion XV, St Louis, MO). Significant differences at P ≤ 0.05 were calculated by ANOVA followed by a Tukey-Kramer test [86].

## Abbreviations

ANOVA - analysis of variance, bp - base pairs, cDNA - complementary DNA, DNA - deoxyribonucleic acid, EST - expressed sequence tag, FT-ICR/MS - Fourier transform-ion cyclotron resonance mass spectrometry, GO - gene ontology, IQL - interquartile length, JGI - Joint Genome Institute, KEGG - Kyoto Encyclopedia of Genes and Genomes, m/z - mass-to-charge ratio, Mya - million years, ORF - open reading frame, *P*. - *Populus*, ppm - parts per million, qRT-PCR - quantitative real-time polymerase chain reaction, REST - relative expression software tool, RMA - robust multi-array average, SAM - significance analysis of microarrays, TAIR - The Arabidopsis Information Resource

## Authors' contributions

DJ conducted this work as part of his PhD thesis. He performed the experiments, did the bioinformatic analyses and drafted the manuscript. PSK and BK provided FT-ICR-MS data and conducted the hierarchical clustering analysis. KB and JPS conducted gas exchange analyses. AP designed the experiments and finalized the study. All authors read and commented on the manuscript.

## Supplementary Material

Additional file 1 **Table S1: List of genes with significantly different signal intensities in *P. euphratica *compared with *P*. × *canescens***. The table contains fold-change of *P. euphratica/P*. × *canescens*, filter settings according to Table 2 in the manuscript and genes present after application of filters. **Table S2: **Mapman analysis of genes with significant differences in transcript levels *P. euphratica *compared with *P*. × *canescens*. The gene list with AGI annotations obtained after filer 5 was used and genes were to the 35 categories present under "Mapman overview". **Table S3: **List of metabolites with significant differences between *P. euphratica *compared with *P*. × *canescens*. Positive peak ratios indicate significant enrichment in *P. euphratica *and negative ratios indicate significant enrichment in *P*. × *canescens*, respectively. **Table S4: **Primers used to determine gene expression by qRT-PCR. Gene names, accession numbers and primer sequences are shown. Click here for file
